# Dosimetric Comparison of Ultra-Hypofractionated and Conventionally Fractionated Radiation Therapy Boosts for Patients with High-Risk Prostate Cancer

**DOI:** 10.3390/life12030394

**Published:** 2022-03-09

**Authors:** Tomasz Piotrowski, Slav Yartsev, Jaroslaw Krawczyk, Marta Adamczyk, Agata Jodda, Julian Malicki, Piotr Milecki

**Affiliations:** 1 Department of Electroradiology, Poznan University of Medical Sciences, 61-866 Poznan, Poland; julian.malicki@wco.pl (J.M.); piotr.milecki@wco.pl (P.M.); 2 Department of Medical Physics, Greater Poland Cancer Centre, 61-866 Poznan, Poland; marta.adamczyk@wco.pl (M.A.); agata.jodda@wco.pl (A.J.); 3 Department of Biophysics, University of Western Ontario, London, ON N6A 5C1, Canada; yartsevslav@gmail.com; 4 Radiotherapy Department I, Greater Poland Cancer Centre, 61-866 Poznan, Poland; j.krawczyk0711@wp.pl

**Keywords:** prostate, ultra-hypofractionated radiation therapy, dosimetric comparison, dose metrics, complexity

## Abstract

Recent comparison of an ultra-hypofractionated radiotherapy (UF-RT) boost to a conventionally fractionated (CF-RT) option showed similar toxicity and disease control outcomes. An analysis of the treatment plans for these patients is needed for evaluating calculated doses for different organs, treatment beam-on time, and requirements for human and financial resources. Eighty-six plans for UF-RT and 93 plans for CF-RT schemes were evaluated. The biologically equivalent dose, EQD2, summed for the first phase and the boost, was calculated for dose-volume parameters for organs at risk (OARs), as well as for the PTV1. ArcCHECK measurements for the boost plans were used for a comparison of planned and delivered doses. Monitor units and beam-on times were recorded by the Eclipse treatment planning system. Statistical analysis was performed with a significance level of 0.05. Dosimetric parameter values for OARs were well within tolerance for both groups. EQD2 for the PTV1 was on average 84 Gy for UF-RT patients and 76 Gy for CF-RT patients. Gamma passing rate for planned/delivered doses comparison was above 98% for both groups with 3 mm/3% distance to agreement/dose difference criteria. Total monitor units per fraction were 647 ± 94 and 2034 ± 570 for CF-RT and UF-RT, respectively. The total delivery time for boost radiation for the patients in the UF-RT arm was, on average, four times less than the total time for a conventional regimen with statistically equal clinical outcomes for the two arms in this study.

## 1. Introduction

Despite being the leading cancer disease for men, the optimal/best choice of prostate cancer (PCa) treatment for a particular patient is still an active area of research [1,2,3]. Improvement of a PCa cure is related to oncological, psychological, and economic issues [4,5,6]. Different fractionation schemes for radiation therapy were explored [7,8,9,10]. Clinical data for low- and intermediate-risk PCa show that treatment outcomes (i.e., genitourinary and gastrointestinal toxicities, biochemical relapse free survival (BRFS), overall survival (OS), and metastases-free survival (MFS)) after ultra-hypofractionated radiation therapy (UF-RT) are similar to conventional-fractionated radiation therapy (CF-RT) [11]. UF-RT for high-risk PCa is still regarded as an experimental method because toxicity and disease control results remain undefined [3]. The gradual introduction of UF-RT in clinics began with assessing this possibility for the boost portion of the treatment. In a recent study, Milecki et al. [12] compared the 5-year tolerance and survival for high-risk PCa patients undergoing a UF-RT boost to those undergoing a CF-RT boost to the prostate gland plus seminal vesicles after whole pelvic CF-RT combined with androgen deprivation therapy (ADT). This study demonstrated that a combination of the UF-RT boost with long-term ADT was well-tolerated and yielded good efficacy results compared to the CF-RT boost with ADT. Automatization by artificial intelligence of the treatment process, including treatment modality choice and adequate planning, requires knowledge of the correlation between patient-specific features, treatment plans, and clinical outcomes [13]. In this communication, we analyze the clinical outcomes in their relation to planning parameters and accumulated doses in the planning target volume and organs at risk for the group of patients included in the study of Milecki et al. [12].

## 2. Materials and Methods

### 2.1. Patient Data

Between January 2012 and December 2016, 208 patients were enrolled in the HYPO-PROST randomized trial with two arms (UF-RT and CF-RT), treated with either fixed beam image-guided (IG) intensity-modulated radiation therapy (IMRT) (29 patients) or rotational volumetric modulated arc therapy (VMAT) (179 patients) [12].

The inclusion criteria were: (1) age of the patients ranged from 45 to 75 years; (2) prostate biopsy performed <180 days prior to randomization; (3) Prostate Specific Antigen (PSA) marked at least 10 days after or prior to biopsy (for patients taking phiansteroid 30 days after stopping it); (4) the presence of at least one of the following parameters-cT3*, Gleason score higher than 7, PSA higher than 20 ng/mL, or the presence of at least two of the following parameters-cT2c, Gleason score equal to 7, PSA in the range from 10.1 to 19.9 ng/mL; (5) minimal three months ADT before RT, followed by long-term ADT up to 24 months; (6) general condition according to the Eastern Cooperative Oncology Group classification scored as 0 or 1; (7) no distant and regional metastases confirmed by skeletal scintigraphy, chest X-ray, pelvic computed tomography or pelvic magnetic resonance; (8) morphological and biochemical parameters within the normal range; and (9) signing the informed consent to participate in the study. The exclusion criteria were: (1) the presence of neoplastic disease, with the exception of skin cancer, less than 5 years prior to randomization; (2) prior radical prostatectomy or radiation therapy in the pelvic area; (3) earlier hormone therapy, other than that indicated in the study protocol; (4) comorbidities that may significantly affect the patient’s life expectancy; and (5) failure to meet the criteria for including the patient in the study.

The trial was approved by Bioethics Committee at Poznan University of Medical Sciences. All patients signed an informed consent to participate in this study.

### 2.2. Treatment Planning and Dose Delivery

This dosimetrical analysis includes only 179 patients treated by the VMAT technique. All patients underwent planning computed tomography (CT). CT images (Definition AS, Siemens, Erlangen, Germany) were reconstructed with 3 mm spacing. The patients were scanned in a supine position with a knee-fix (CIVCO Radiotherapy, Coralville, IA, USA) immobilization system. At least seven days before the CT imaging, patients were given fiducial markers (Gold AnchorTM; Naslund Medical AB, Huddinge, Sweden) applied to the prostate gland under the control of a transrectal ultrasound. Thirty minutes before the CT imaging, the patients were asked to empty their bladder and then to drink 0.5 L of water. Neither an endorectal balloon nor pharmacological or mechanical preparations were used for the rectum. The gross tumor volume (GTV) was defined as the total volume of the prostate gland along with the basal volume of the seminal vesicles defined within 1 cm from the prostate gland. Two clinical target volumes (CTV) were: (1) the CTV1 defined as the GTV plus a 5 mm margin, except for the rectum interface, where the margin was 0 mm; and (2) the CTV2 defined as the CTV1 plus pelvic lymphatic system (obturator nodes, pre-sacral nodes, internal iliac nodes, and external iliac nodes) [14]. Planning target volumes (PTV) were defined as CTV2 plus 5 mm margin (PTV2) and CTV1 plus 3 mm margin (PTV1). The following organs at risk (OARs) were delineated for each patient: rectum, bladder, bowels, and left and right femoral heads. The rectum was contoured from the anus to the sigmoid flexure. The bowels were contoured from the L4-5 interspace to its lowest extent in the pelvis as an entire bag. The outlines were performed by a group of three radiation oncologists with more than twenty years of experience who specialize in genitourinary carcinomas.

The first phase of RT provided irradiation of the PTV2 to the total dose of 46 Gy using 2 Gy fractionation. In the second phase, the irradiated target was PTV1. According to the randomization, 93 patients were allocated in the conventionally fractionated radiation therapy (CF-RT) arm. They received a boost of 30 Gy (15 fractions × 2 Gy). The other 86 patients were allocated to the ultra-hypofractionated radiation therapy (UF-RT) arm and received a boost of 15 Gy (2 fractions × 7.5 Gy) within ten days. The treatment plans were prepared for a Varian TrueBeam™ accelerator (Varian Medical Systems, Palo Alto, CA, USA) using the Eclipse™ treatment planning system ver. 13.6 (Varian Medical Systems, Palo Alto, CA, USA). The analytic anisotropic algorithm with a spatial resolution of 2.5 mm was used for computing the dose to the irradiated region. For each of 179 patients, 6 MV 2-arc VMAT treatment plans for the first phase of irradiation were prepared. In the second phase, the same scheme was used for the 93 patients allocated to CF-RT arm. For the 86 patients from the UF-RT arm, the final number of arcs in the second phase depended on the optimization result, i.e., the initial plan assumed the use of two arcs, if such a number of arcs did not guarantee the achievement of optimization goals, the number of arcs was increased. As result, two arcs were used for six patients, three arcs for 71 patients, and four arcs for nine patients. Table 1 shows general information about the radiotherapy schemes used in the study. The physical doses obtained from non-conventional fractionations (e.g., the second phase of UF-RT) were recalculated to biologically equivalent doses using a EQD2 formula based on the linear quadratic model [15,16]. The recalculations were performed in Velocity^TM^ software (Varian Medical Systems, Palo Alto, CA, USA). The α/β ratios used in the recalculations were 1.5 for the tumor and 3.0 for the OARs [17]. Before the plan acceptance for delivery, the biologically equivalent doses from the first and second phases were summed and checked for adherence to the required dose constraints for the OARs (Table 2), as well as to adequate dose distribution in the PTVs. The ICRU-83 plan normalization criteria for the PTVs were followed, with prescription to the median dose [18]. The treatment plans were performed by a group of five medical physicists with more than fifteen years of experience each [19].

Before dose delivery to the patient, the dosimetric verification for every plan was performed by an evaluation of the agreement between the planned and delivered doses. The ArcCHECK cylindrical diode array (Sun Nuclear Inc, Melbourne, FL, USA) was used for pre-treatment dosimetric verification. The treatment-planned dose distributions were re-calculated on the ArcCHECK CT (Computed Tomography) (dose grid size: 2.5 mm) for the actual planned gantry and collimator angles. The SNC software ver. 8.2.0 (Sun Nuclear Inc, Melbourne, FL, USA), integrated with the ArcCHECK hardware, was used for the evaluation based on the gamma method. The evaluation was performed in a global mode for a routinely used combination of γ-index criteria, i.e., 3% of dose difference (DD) and 3 mm of distance to agreement (DTA). The threshold of the values of the analysed doses was 10% and was normalised to the maximum planned dose. The plans where the gamma passing rate (GPR) was higher than 95% were considered acceptable.

During the treatment realization, the fraction dose irradiation was preceded by daily IG procedures. In the first phase of treatment, and the second phase in the CF-RT arm, IG based on kilo-electron volt cone-beam computed tomography (keV-CBCT) images were used to check the position of the prostate, including its relation to OARs (first day of the week), and two, orthogonal two-dimensional kilo-electron volt (2D-keV) images were used to quickly check the position of fiducial markers implemented in the prostate (next four days of the week). For the second phase of the UF-RT arm, only IG based on keV-CBCT images were used.

### 2.3. Data Comparison and Statistical Analysis

The treatment plans for the CF-RT and UF-RT arms were analyzed for the potential differences between dose metrics, the complexity of the plans, and the agreement between planned and delivered doses, using the following parameters:

(a) Dose metrics
  ⇒ The mean EQD2 [Gy] and the EQD2 related to 2% (D2 [Gy]) and 95% (D95 [Gy]) of the PTV1 (the prostate and the basal volume of seminal vesicles);
  ⇒ The EQD2 related to 5% (D5 [Gy]), 25% (D25 [Gy]), 30% (D30 [Gy]), and 40% (D40 [Gy]) of the rectum volume;
  ⇒ The EQD2 related to 10% (D10 [Gy]), 25% (D25 [Gy]), 30% (D30 [Gy]), and 40% (D40 [Gy]) of the bladder volume;
  ⇒ The EQD2 related to 10% (D10 [Gy]) of the volumes of femoral heads and the bowels.

The parameters of EQD2 correspond to the dose constraints used in the study (Table 2) and were composed of the sum of the doses obtained from the first and second phases of irradiation.

(b) Complexity of the plans
  ⇒ The mean number of monitor units per control point (MMU [MU]);
  ⇒ The mean dose rate per control point (MDR [MU/min]);
  ⇒ Total monitor units per fraction [MU];
  ⇒ Delivery time per fraction [min].
(c) Agreement between planned and delivered doses
  ⇒ The score of gamma passing rate in percent (GPR [%]) obtained from gamma analysis performed in a global mode, using a 3%/3 mm γ-index criteria and a 10% threshold.

The parameters for the complexity of the plans, as well as for agreement between planned and delivered doses, were evaluated for the second phase of irradiation only.

The evaluation of each parameter started by checking the normality of the distribution (by the Shapiro–Wilk test), and the homogeneity of the variances between the compared groups (by the Fisher F-test). If the normality and homogeneity of the variances were established, the parametrical, two-tailed, t-Student test for independent samples was used to compare mean values of parameters obtained for the CF-RT and UF-RT arms. In other situations (normality and/or homogeneity not confirmed), the non-parametrical Mann–Whitney test for independent samples was used. The correlations between gamma passing rates and the complexity indices were checked by the Spearman method. All tests were performed with the significance level equal to 0.05.

## 3. Results

Table 3 shows the results of the statistical comparison for parameters of dose metrics in specified parts of structures that were obtained from the summed doses of the first and second phases of irradiation in the CF-RT and UF-RT arms.

The statistical differences between the mean values of EQD2 parameters obtained for PTV1 (Table 3) are caused by the scheme of treatment (Table 1). Despite the increase in the dose delivered to PTV1 (e.g., mean EQD2 for UF-RT equal to 84.6 Gy vs. 76.3 Gy for CF-RT; *p* < 0.001), the doses delivered to the OARs were clinically comparable (Table 3). Due to the clinical constraints (Table 2), the statistical superiority of UF-RT for the doses D25, D30, and D40 of the rectum, and D10 of the femoral heads (*p* < 0.001 for each comparison), were not clinically relevant.

While the doses in the OARs, in compared arms of treatment (CF-RT vs. UF-RT), were clinically comparable, the parameter values describing the complexity of the second phase of treatment were significantly higher for the UF-RT arm (Table 4).

**Figure 1:**
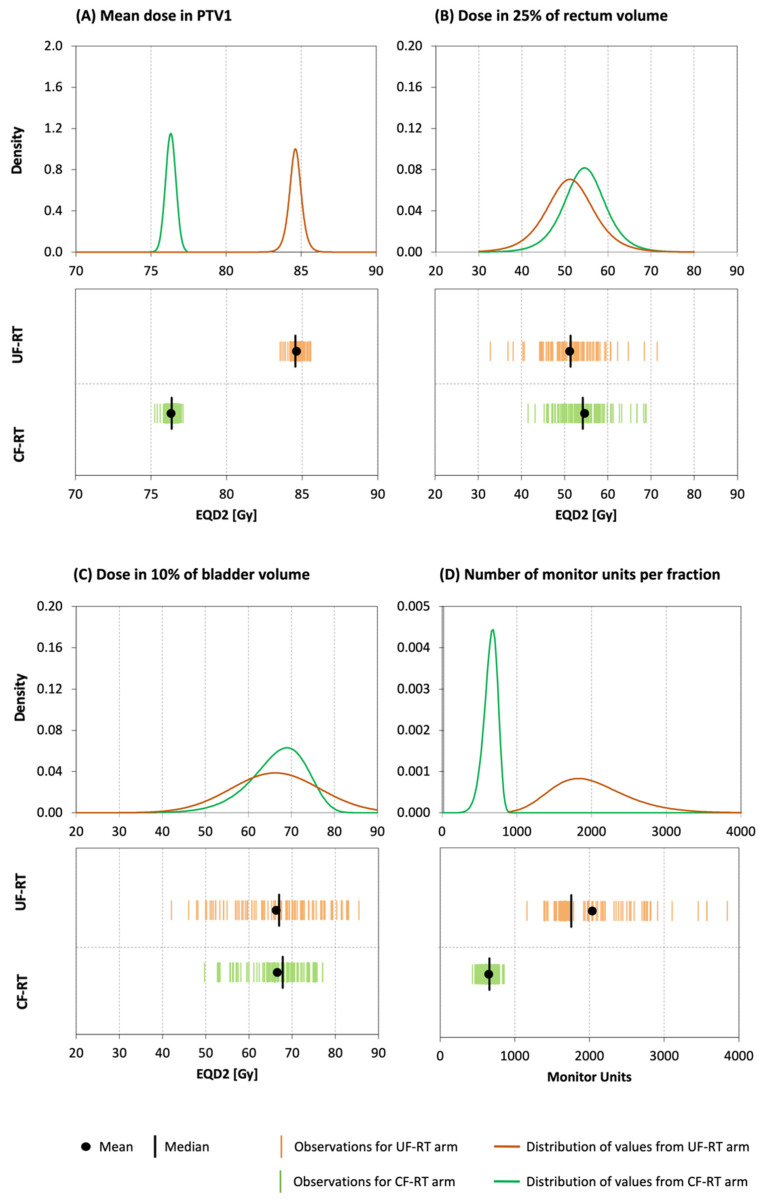
EQD2 values obtained for the boost in CF-RT and UF-RT arms for selected structures and dose metrics: (**A**) mean dose in PTV1; (**B**) D25 in the rectum; (**C**) D10 in the bladder; and (**D**) total monitor units per fraction (TMU). Upper graphs show the density of the parameters’ distribution, and the lower panels show their spread along the x-axis.

Figure 1 shows results obtained for (A) mean dose in PTV1, (B) D25 in the rectum, (C) D10 in the bladder, and (D) total monitor units per fraction (TMU) in the second phase of irradiation. The mean doses in PTV1 are characterized by a normal distribution, homogeneity of the variances, and statistically (and also clinically) significant differences between the values for the two arms (Table 3). The doses to 25% of the rectum are also characterized by a normal distribution and homogeneity of variances. Nevertheless, the statistically significant differences between the values (i.e., 51.2 Gy for UF-RT vs. 54.6 Gy for CF-RT; *p* < 0.001) are not clinically relevant (i.e., both values are much lower than the dose constraint of 70 Gy required in study; Table 2). While the distributions of D10 in the bladder were normal for both arms (*p* = 0.220 for UF-RT and *p* = 0.165 for CF-RT), the comparison shows a significant difference between their variances (*p* < 0.001), and no significant difference between their means (*p* = 0.980). The distributions, as well as homogeneities of the TMU in the compared arms, were statistically different (Table 4). The mean of the TMU was significantly higher (*p* < 0.001) for UF-RT (2034.3 MU) than for CF-RT (647.3 MU).

**Figure 2:**
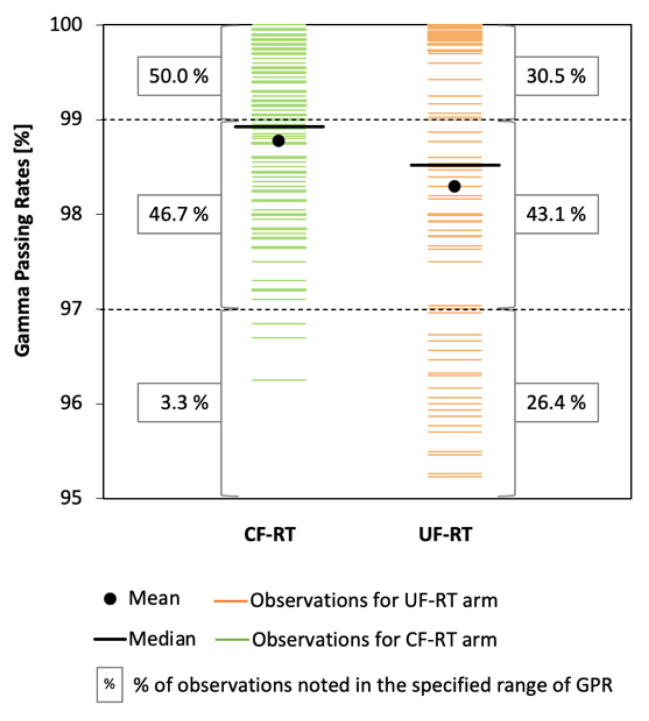
Gamma passing rates for the CF-RT and UF-RT arms.

Although UF-RT plans had a greater complexity than CF-RT plans, no significant differences were found between the results of agreement between planned and delivered doses for the compared arms (*p* = 0.219; Table 4). It should be noted, however, that the CF-RT arm had a higher percentage of better results. For example, GPR > 99% was obtained for 50% of observations in CF-RT, while for UF-RT it was only 30.5% of observations (Figure 2).

**Figure 3:**
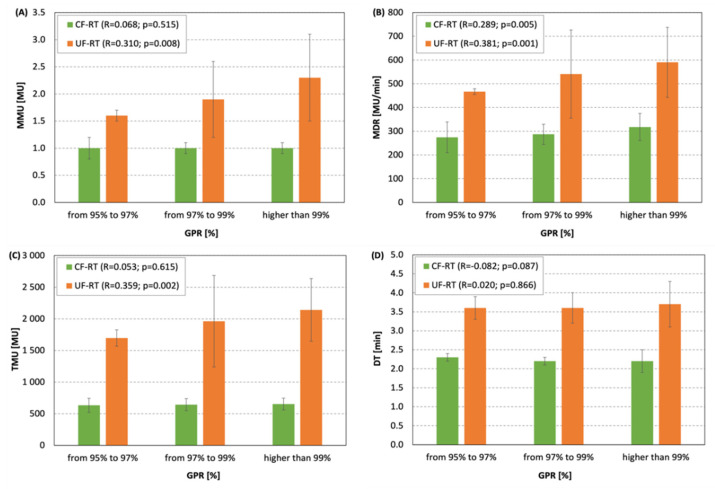
Correlations between the ranges of gamma passing ratios (GPR) and (**A**) mean monitor units per control point (MMU), (**B**) mean dose rate per control point (MDR), (**C**) total monitor units per fractions (TMU), and (**D**) delivery time per fraction (DT). Correlations for the CF-RT and UF-RT arms were performed separately using the Spearman method at a 0.05 significance level.

The average GPRs were found to depend on the number of arcs used in the UF-RT arm: 99.8% for 2 arcs, 98.1% for 3 arcs, and 98.6% for 4 arcs. A weak, but statistically significant, correlation between GPRs and MMU, MDR, and TMU indices was detected for the UF-RT arm (Figure 3).

## 4. Discussion

A dosimetric comparison of the results obtained for the two arms of radiotherapy treatment (CF-RT and UF-RT) for high-risk PCa patients is important to establish factors that influence clinical outcomes. The cumulative incidence of gastrointestinal grade 2 or worse late toxicities was 13.9% for UF-RT vs. 8.6% for CF-RT; HR 1.75 (95% CI 0.76–5.05); *p* = 0.180, and for genitourinary grade 2 or worse late toxicities was 5.9% for UF-RT vs. 5.8% for CF-RT; HR 1.06 (95% CI, 0.34–3.30); *p* = 0.910, respectively. Although we observed no statistically significant differences in late toxicities between UF-RT and CF-RT, a slight trend for higher gastrointestinal toxicity for UF-RT indicates the necessity to pay more attention to these organs in patient bowel preparation and pre-treatment imaging for such patients.

One of the study’s main limitations was no access to the technologies of dose-guidance (DG) procedures during the treatment (at that time) in our hospital. The DG procedures provide information on delivered doses based on dose reconstruction on CBCT images. Giacometti et al. [20] show the currently used DR procedures based on CBCT. Despite continuous improvements in the technology used to create CBCT images, direct calculations of dose distributions on CBCT images are still subject to a greater error than calculations made on CT images [21]. One of the methods to reduce inaccuracies of dose recalculation is based on the use of CBCT images to transform the CT images used in planning. The dose, in this method, is recalculated on transformed CT images [22]. Unfortunately, the prospective nature of our study, and the lack of availability to clinically approved DG procedures in our hospital in the years from 2012 to 2016, led to image guidance procedures being used during the treatment only. We believe that information from DR procedures can link our plan dosimetry data with the treatment toxicity data more appropriately. Therefore, we plan to explore delivered dose reconstruction using deformable image registration (DIR) of the CBCT studies in the future.

In general, plan dosimetry studies are limited by the nature of compared data that represent a computer simulation of physical dose delivery, based on imaging from a snapshot in time several days before actual delivery. When the on-screen plan is delivered by a treatment unit, there will be some degradation based on the complexity of the plan and the performance of the equipment [23]. Therefore, except for the analysis of dose metrics, the complexity of the treatment plans and parameter describing an agreement between the planned and delivered doses were included in our analysis. Several indices for the characterization of plan complexity were proposed [24], and we used the ones that are easy to extract from the Eclipse treatment planning system: mean monitor unit and mean dose rate per control point [25]. We also analyzed total monitor unit and beam-on delivery time per fraction.

Our results show that the UF-RT scheme offered higher EQD2 in PTV1 with the OAR doses similar to those for the CF-RT regimen. The UF-RT plans of the second phase of treatment are characterized by higher complexity than the plans with conventional fractionation (CF-RT). For example, most of the plans (83%) for the patients in the UF-RT arm needed 3 arcs and the mean monitor units per control point, as well as total monitor units per fraction, were twice as large for the UF-RT plans than for the CF-RT plans (Table 4). While better results were observed for CF-RT, the results for both arms were clinically acceptable (GPR > 95%; Figure 2) and allowed for the realization of the plans in the clinic. Interestingly, our analysis of the details of the ArcCHECK results for the UF-RT arm showed that a better agreement between the planned and delivered doses was observed for six plans with 2 arcs with GPR = 99.8%, and that low values of MMU, MDR, and TMU led to worse results for GPR (Figure 3). Since total beam-on time for UF-RT is not affecting the planned/delivered doses agreement, we hypothesize that avoiding small values of monitor units per control point may improve the accuracy of radiation delivery. No statistically significant differences in late toxicities between the UF-RT and CF-RT arms, in addition to the similarity between 5-years of overall survival, metastasis-free survival, and biochemical relapse-free survival gathered from clinical outcomes [12], are in line with our dosimetry findings about similarity between the UF-RT and CF-RT plans in dosimetric parameters, plan complexity, and deliverability of the plans.

A higher biologically equivalent dose (84 Gy) achieved for the PTV1 in the plans for UF-RT patients compared to 74 Gy in CF-RT plans was expected to result in a better tumoricidal effect [26]. However, our clinical outcomes data showed no obvious advantage in this respect [12] and we continue to explore this question.

The data in Table 4 shows that the total time for boost radiation delivery for the patients in the UF-RT arm was, on average, four times less than the total time for the conventional regimen (i.e., 2 fractions × 3.6 min vs. 15 fractions × 2.2 min). This is an important advantage because the possibility of the target moving, as well as the discomfort of the patient, increases with greater time spent during treatment. The less total monitor units (more than 2 times, i.e., 2 fractions × 2034 MU vs. 15 fractions × 647 MU) needed for treatment of the UF-RT patients saves energy and equipment wear. Finally, two sessions of boost treatments are preferable for the patient to 15, both physically and psychologically.

This study is only a single step on the way to personalized radiotherapy of high-risk prostate cancer patients that includes the following: specific patient data, diagnostic imaging options, choice of treatment modality, treatment plan, quality assurance, pre-treatment image guidance, immobilization devices, plan adaptation, acute toxicity, and long-term clinical outcomes. An evaluation of each step based on available technology, and human and financial resources is needed. We started with a dosimetric study of the treatment plans for 179 patients enrolled in the study [12]. Including more parameters (patient’s age, body-mass index, PTV1, PTV2, anatomical features, and genetic data) might require a larger cohort of patients to achieve sufficient statistical power for predictions.

## 5. Conclusions

A dosimetric analysis of the boost plans prepared for 7.5 Gy × 2 (UF-RT) and 2 Gy × 15 (CF-RT) schemes showed that biologically equivalent doses EQD2 for organs at risk were statistically close for both schemes, which is in agreement with clinically observed similar toxicity outcomes for these patient groups. If α/β = 1.5 is used for calculation of EDQ2 for the prostate gland, the sum of EQD2 for two phases were 84 and 76 Gy for the UF-RT and CF-RT arms, respectively. A good agreement between planned and delivered doses was found by ArcCHECK measurements for all plans. The UF-RT scheme is preferable for saving human and financial resources due to a shorter total irradiation time and less total monitor units.

## Figures and Tables

**Table 1:** Radiotherapy schemes used in the study.

	CF-RT		UF-RT	
Number of patients	93		86	
Phase of irradiation	1	2	1	2
Treatment volume	PTV2 (PG + SV + LN)	PTV1 (PG + SVbase)	PTV2 (PG + SV + LN)	PTV1 (PG + SVbase)
Physical Dose [Gy]	46	30	46	15
Number of fractions	23	15	23	2
EQD2 [Gy] in PTV1	76		84	
Fractionation scheme	5 fractions per week, i.e., one fraction per day with gap on Saturday and Sunday			2 fractions received within ten days
Technique, source, and energy	VMAT X 6 MeV			
Number of arcs per plan (% of patients)	2 (100%)			2 (7%)
				3 (83%)
				4 (10%)

PG, prostate gland; SV, seminal vesicles; SVbase, base of seminal vesicles; LN, lymph nodes.

**Table 2:** The dose constraints for organs at risk used during optimization.

Structure	% of Volume				
	5%	10%	25%	30%	40%
	Dose Constraints [Gy]				
Rectum	≤75	---	≤70	≤60	≤50
Bladder	---	≤75	≤70	≤60	≤50
Femoral heads	---	≤50	---	---	---
Bowels	---	≤40	---	---	---

**Table 3:** Statistical analysis of dose metric parameters in specified parts of structures, obtained from the summed doses of the first and second phases of irradiation in the CF-RT and UF-RT arms.

Structure	Parameter	Scheme	EQD2 [Gy]	Normality	HoV	SoM
			Mean (SD)	Shapiro-Wilk Test	Fisher’s F-Test	t-Student (tS) or Mann-Whitney (MW)
PTV1	D95	CF-RT	74.7 (0.4)	*p* = 0.988	*p* < 0.001	*p* < 0.001 (MW)
		UF-RT	80.7 (0.7)	*p* = 0.532		
	Mean Dose	CF-RT	76.3 (0.3)	*p* = 0.092	*p* = 0.064	*p* < 0.001 (tS)
		UF-RT	84.6 (0.5)	*p* = 0.098		
	D2	CF-RT	78.3 (0.4)	*p* = 0.976	*p* = 0.090	*p* < 0.001 (tS)
		UF-RT	87.9 (0.5)	*p* = 0.147		
Rectum	D5	CF-RT	72.4 (2.8)	*p* = 0.068	*p* < 0.001	*p* = 0.953 (MW)
		UF-RT	71.9 (6.6)	*p* = 0.170		
	D25	CF-RT	54.6 (5.5)	*p* = 0.148	*p* = 0.177	*p* < 0.001 (tS)
		UF-RT	51.2 (6.4)	*p* = 0.134		
	D30	CF-RT	51.9 (5.3)	*p* = 0.375	*p* = 0.108	*p* < 0.001 (tS)
		UF-RT	48.0 (6.3)	*p* = 0.066		
	D40	CF-RT	47.4 (5.3)	*p* = 0.364	*p* = 0.603	*p* < 0.001 (tS)
		UF-RT	43.5 (5.0)	*p* = 0.072		
Bladder	D10	CF-RT	66.6 (6.9)	*p* = 0.165	*p* < 0.001	*p* = 0.980 (MW)
		UF-RT	66.3 (9.3)	*p* = 0.220		
	D25	CF-RT	50.6 (8.6)	*p* = 0.153	*p* = 0.691	*p* = 0.633 (tS)
		UF-RT	50.0 (8.9)	*p* = 0.085		
	D30	CF-RT	46.8 (8.3)	*p* = 0.325	*p* = 0.582	*p* = 0.540 (tS)
		UF-RT	46.0 (8.8)	*p* = 0.266		
	D40	CF-RT	40.2 (7.8)	*p* = 0.512	*p* = 0.594	*p* = 0.501 (tS)
		UF-RT	39.4 (8.2)	*p* = 0.295		
Left FH	D10	CF-RT	38.9 (3.8)	*p* = 0.571	*p* = 0.865	*p* < 0.001 (tS)
		UF-RT	34.6 (3.7)	*p* = 0.257		
Right FH	D10	CF-RT	38.5 (4.2)	*p* = 0.426	*p* = 0.529	*p* < 0.001 (tS)
		UF-RT	34.5 (3.9)	*p* = 0.616		
Bowels	D10	CF-RT	36.9 (5.4)	*p* = 0.166	*p* = 0.750	*p* = 0.659 (tS)
		UF-RT	37.3 (5.3)	*p* = 0.054		

HoV, homogeneity of the variations; SoM, similarity of the means; SD, standard deviation; FH, femoral head.

**Table 4:** Statistical analysis for complexity indices and gamma passing rates in the UF-RT and CF-RT arms.

Parameter	Scheme	Mean (SD)	Normality	HoV	SoM
			(Shapiro-Wilk Test)	(Fisher’s F-Test)	(Mann-Whitney Test)
Mean monitor units per control point [MU]	CF-RT	1.0 (0.1)	*p* = 0.495	*p* < 0.001	*p* < 0.001
	UF-RT	2.1 (0.7)	*p* < 0.001		
Mean dose rate per control point [MU/min]	CF-RT	300.7 (52.6)	*p* = 0.222	*p* < 0.001	*p* < 0.001
	UF-RT	570.6 (171.9)	*p* < 0.001		
Total monitor units per fraction [MU]	CF-RT	647.3 (94.0)	*p* = 0.225	*p* < 0.001	*p* < 0.001
	UF-RT	2034.3 (570.3)	*p* < 0.001		
Delivery time per fraction [min]	CF-RT	2.2 (0.2)	*p* < 0.001	*p* < 0.001	*p* < 0.001
	UF-RT	3.6 (0.5)	*p* = 0.002		
Gamma passing rate [%]	CF-RT	98.8 (0.9)	*p* = 0.001	*p* < 0.001	*p* = 0.219
	UF-RT	98.3 (1.5)	*p* < 0.001		

HoV, homogeneity of the variations; SoM, similarity of the means; SD, standard deviation.

## Data Availability

The datasets analyzed during the current study are available from the corresponding author upon request.
